# Novel homozygous frameshift insertion variant in the last exon of the *EDARADD* causing hypohidrotic ectodermal dysplasia in two siblings: case report and review of the literature

**DOI:** 10.1186/s13052-024-01681-2

**Published:** 2024-06-05

**Authors:** Ahmet Kablan, Elifcan Tasdelen

**Affiliations:** 1 Department of Medical Genetics, Sanliurfa Research and Training Hospital, Sanliurfa, Turkey; 2 Department of Medical Genetics, Etlik City Hospital, Ankara, Turkey

**Keywords:** HED, *EDARADD*, Insertion, Novel variant, Case report

## Abstract

**Background:**

Hypohidrotic ectodermal dysplasia (HED) is a genetic disorder that results in the abnormal development of structures derived from ectodermal tissue. This rare condition predominantly affects the hair, nails, eccrine glands, and teeth. While HED can be caused by various genes, the *EDA, EDAR, EDARADD*, and *WNT10A* genes account for approximately 90% of cases. Notably, HED forms associated with variants in the *EDA, EDAR*, or *EDARADD* genes may exhibit similar phenotypes due to defects in a common signaling pathway. Proper interaction among the products of these genes is crucial for the activation of the nuclear factor (NF-κB) signaling pathway, which subsequently regulates the transcription of targeted genes. The *EDARADD* gene, in particular, harbors one of the rarest reported variants associated with HED.

**Case presentation:**

Five-and two-years-old brothers born into consanguineous parents were examined at our outpatient medical genetics clinic at Sanliurfa Training and Research Hospital, Turkey. Both displayed the same classical phenotypic features of HED. The elder had a very sparse dark and brittle hair, sparse eyebrows and eyelashes, conical upper and lower premolar teeth with hypodontia, widely spaced teeth, very dry skin, mildly prominent forehead, and periorbital wrinkles. The younger one showed the same, but less severe, clinical features. After thorough examination and patient history evaluation, targeted next-generation sequencing analysis yielded the novel homozygous insertion variant c.322_323insCGGGC p.(Arg108ProfsTer7) in *EDARADD.* The mutation has not been reported to date in the literature.

**Conclusions:**

In this report, we present two siblings exhibiting classical HED symptoms and a novel insertion variant of the *EDARADD* gene, which leads to a frameshift introducing a stop codon. Both brothers inherited such mutation from their parents, who were heterozygous carriers of the same variant. The present study may shed light about the pathogenic mechanisms underlying HED, and expand the spectrum of *EDARADD* gene variants associated with this condition.

**Supplementary Information:**

The online version contains supplementary material available at 10.1186/s13052-024-01681-2.

## Background

Ectodermal dysplasia (ED) is a genetically heterogeneous condition characterized by the abnormal development of structures derived from the ectodermal tissue [1]. Among its most frequent forms there are hypohidrotic and anhidrotic ectodermal dysplasias (HED/AED), which present a triad of symptoms, including scalp alopecia or sparse hair (generalized hypotrichosis), teeth anomalies, and hypohidrosis. In addition, some patients may also exhibit minor dysmorphic features such as forehead bumps, rings under the eyes, everted nose, and prominent lips. Several genes have been implicated in the pathogenesis, with the most commonly identified genetic aetiologies being X-linked *EDA* mutations, as well as autosomal *EDAR* and *EDARADD* mutations. Proper function of these three genes and their products is crucial for downstream activation of the nuclear factor (NF-κB), which subsequently regulates the expression of various genes involved in ectodermal development [2].

*EDARADD* is a recently identified gene that encodes a protein with a death domain in the C-terminus, causing HED with an unclear mechanism [3]. Indeed, the number of reported variants in *EDARADD* remains relatively small, and the majority of these variants are single nucleotide variations. We report on a novel insertion pathogenic variant causing a frameshift of the *EDARADD* gene [4–7]. This variant has been identified, in the homozygous state, in two male siblings born to healthy consanguineous parents. To the best of our knowledge, ours are among the very few patients reported in the literature being affected by HED associated with a pathogenic variant of the *EDARADD* gene.

## Case Presentation

**Fig. 1:**
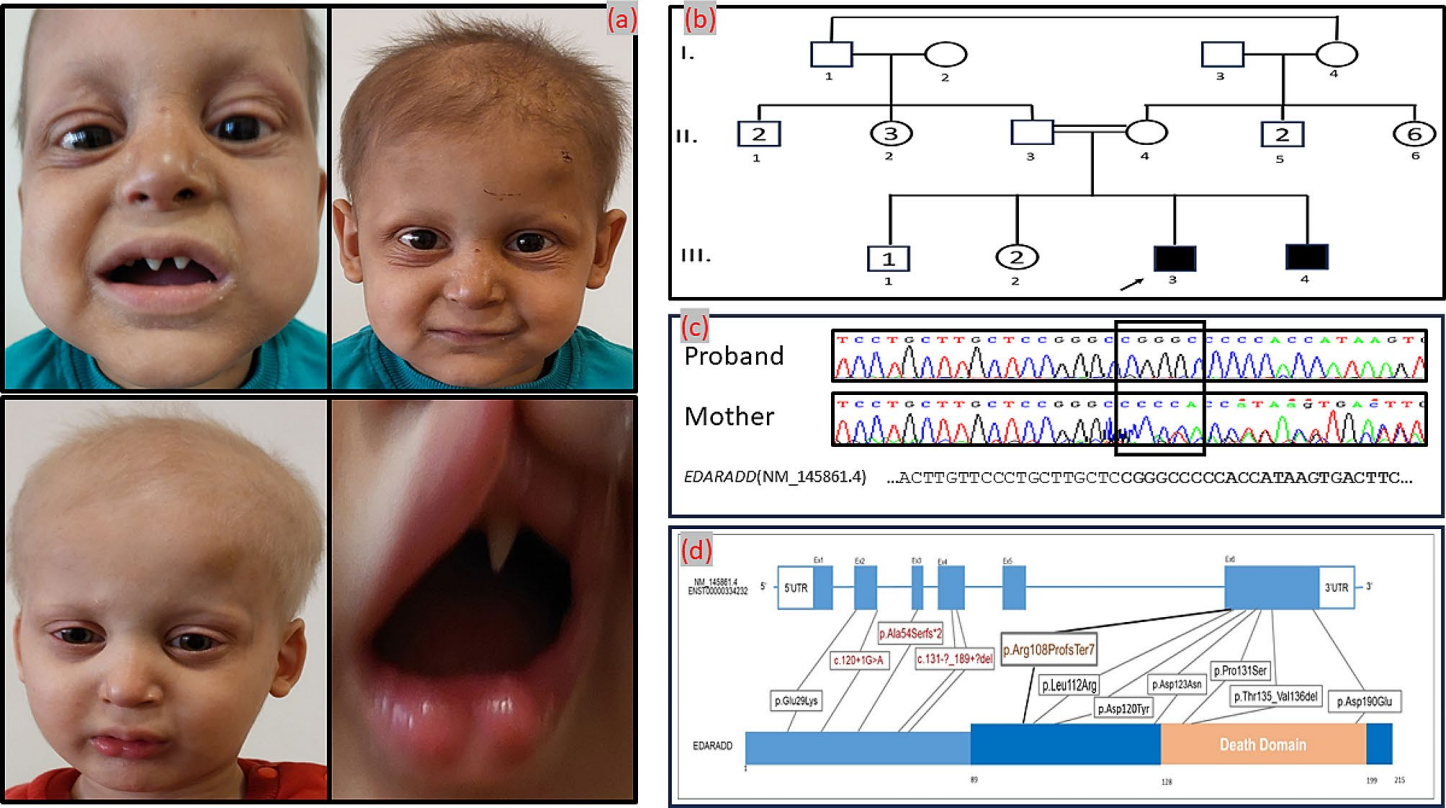
Pictures of affected individuals, pedigree, sequencing display, and representation of reported variants respectively (**a**) Dysmorphic features of the proband (above) and affected younger brother (below). Note the very sparse and brittle hair, absent eyebrows and eyelashes, conical teeth with hypodontia, mildly prominent forehead, and periorbital wrinkles (**b**) Pedigree of the family (**c**) Sanger sequencing display of the proband and heterozygous mother, flanking sequence of the variant. Black rectangle appoints the position of insertion (**d**) Schematic diagram of reported variants. Truncating variants written in red, non-truncating variants in black and the reported variant in brown. Protein region encoded by exon 6 magnified and death domain highlighted with different colour. Note the reported variants position in 5’ upstream of death domain in exon 6

Five- and two-year-old brothers were born uneventfully, into consanguineous (first-degree cousins) parents originating from Syria (Fig. 1b). Both showed the same classical phenotypic features of HED. The elder brother presented with very dry skin, very sparse dark and brittle hair, sparse eyebrows and eyelashes, mildly prominent forehead, periorbital wrinkles, conical upper and lower premolar tooth with hypodontia, and widely spaced teeth (Fig. 1a). His developmental milestones and mental status were normal. The younger sibling displayed slightly milder symptoms, with very sparse blonde hair, a mildly prominent forehead, and teeth anomalies similar to those of the elder brother (Fig. 1a), for which both were receiving dental treatment. The family reported that both brothers have been unable to sweat since birth, and that they had experienced hyperpyrexia due to common infections, successfully managed with medications during hospitalizations. Owing to their reduced ability to sweat, the parents ensured the siblings’ protection from sun by sunscreen use. Notably, both parents denied having any relatives with a similar condition, and further examination confirmed that the remaining three living siblings (1 male, 2 females), and the parents themselves, showed no signs of HED.

Informed consent was obtained from the parents. The *EDA, EDAR, WNT10A*, and *EDARADD* genes were sequenced through NGS following manufacturer’s instructions. Thereafter, Sanger sequencing was conducted, confirming the identified variant, in all family members.

Next-generation sequencing (NGS) analysis did not reveal any causative variants in the *EDA*, *EDAR*, or *WNT10A* genes. However, in the *EDARADD* gene (RefSeq accession number NM_145861), a homozygous pentanucleotide insertion, c.322_323insCGGGC (p.Arg108ProfsTer7), leading to a frameshift which introduces a premature stop codon, was identified and considered likely pathogenic in both siblings, based on the American College of Medical Genetics criteria (PVS1-Pathogenic criterion for predicted loss of function variants, PM2-Population data, PP1-Segregation data). This variant was absent in the Exome Aggregation Consortium and dbSNP database. Subsequent Sanger sequencing was performed in all family members, confirming the homozygosity status in the affected patients. Co-segregation analysis revealed that both parents and one health male sibling were heterozygous carriers of the same mutation (Fig. 1c).

## Discussion and conclusions

Ectodermal dysplasia is a genetically and phenotypically heterogeneous condition characterized by the abnormal development of ectodermal derived structures. Although there have been a few other genes reported in individuals with ED-related findings, for hypohidrotic ectodermal dysplasia (HED) four main genes, namely *EDA* (the most common), in addition to *EDAR, WNT10A*, and *EDARADD*, have been suggested to be causative [8–11]. Neonatologists and paediatricians should raise the suspicion of HED in the presence of congenital skin defects, keeping in mind that such disorder may also be linked with mutations different than those affecting the *EDA* gene, including *EDARADD* [12]. Moreover, both recessive and dominant forms of HED caused by *EDARADD* mutations are clinically indistinguishable, and therefore in these cases genetic investigations are decisive for diagnosis confirmation [3, 7, 13]. Indeed, our patients exhibited the triad of symptoms characteristic of HED, including very sparse and brittle hair, teeth anomalies and reduced sweating. Finally, clinicians have to perform a careful differential diagnosis, including conditions sustained by different genetic aetiology such as mutation in *TP63* [14].

In our study, we conducted next-generation sequencing of *EDARADD*, and identified a homozygous variant c.322_323insCGGGC p.(Arg108ProfsTer7) causing a frameshift, and located 5’ upstream of the death domain in exon 6. Such variant has not been previously reported in HED individuals, and data from large population studies is insufficient to assess its frequency. The mutation is an insertion of 5 bases at position c.322, and is expected to impact the protein, made by 215 amino acids in its wild type, introducing a premature termination codon at position 108.

Further studies confirmed co-segregation of the variant in the parents, and identified the heterozygous state in a healthy brother. Based on the segregation analysis, we concluded that this variant causes the recessive form of the disease, as no other family member exhibited symptoms related to HED.

To date, ten different mutations in *EDARADD* have been associated with HED, with only three of these being deletion or insertion [4, 5, 7, 8, 15–19], and different possible molecular pathogenic mechanisms have been hypothesized (disruption of the interaction with EDAR; impairment of the wild-type EDARADD ability to activate NF-kB, disturbance of the multimerization of EDARADD). Additionally, a homozygous gross deletion c.131-?_189+?del was reported with a loss-of-function mechanism.

Zygosity-position-mutation type does not seem to consistently predict the severity of the condition. Published literature is not conclusive in regard to the pathogenic mechanism of *EDARADD*, probably due to very low number of reported variants. However, the loss-of-function mechanism has been considered as a causing process of the disease linked to *EDARADD* [16, 17]. This may be due to the loss of protein through nonsense mediated mRNA decay (NMD), leading to the production of truncated proteins that are missing the death domain in exon 6 (highly conserved among species, suggesting its essential function) [20].

Three previous reported variants causing premature termination codons, as shown in Table 1, are predicted to go NMD. Since the present variant is not predicted to go NMD but removes more than 10% of transcript, this might be the first truncating variant causing termination codon and not going NMD. Therefore, the predicted molecular pathogenic mechanism is the translation of mRNA containing a premature stop codon, and the production of truncated proteins that are missing the death domain in exon 6. The reported variant is located in the 5’ upstream sequence of the death domain (Fig. 1d), probably leading to its deficient or absent translation. Also, the phenotypic variability of the syndrome may be explained by different conditions such as modifier genetic variations, possible interactions with regulatory factors and/or the involvement of epigenetic mechanisms in addition to *EDARADD* pleiotropy, which has already been described in the literature regarding other genes [21, 22].

**Table 1:** Reported variants, variant types and references in *EDARADD* with the transcript NM_145861 by their position on transcript. Homozygotes written in bold, reported variant is in bold and italic. Last column shows phenotypic features of reported probands

Variant	Variant type	References	Clinical Phenotype
**c.85 G > A; p.Glu29Lys**	Missense	“Ahmed 2021[9]”	Silky sparse hair, Hypohidrotic sweating, normal nails, no skin symptoms, peg shaped teeth, no oligodentia
**c.120 + 1G > A (IVS2 + 1G > A)**	Splice site	“Chaudhary 2016[15]”	Sparse hair and teeth, absent eyebrows, dry and shiny hands, and peg-shaped teeth
c.157insA; p.Ala54Serfs*2	Insertion (frameshift)	“Koguchi-Yoshioka 2015[17]”	No hair and teeth anomalies, no skin lesions, anhidrosis
**c.131-?_189+?del**	Gross deletion	“Cluzeau 2018[16]”	Sparse hair, oligodontia with conical and widely spaced teeth, ichthyosiform dry skin, anhidrosis, prominent lips, frontal bossing, pointed chin, recurrent pulmonary infections
***c.322_323insCGGGC p.Arg108ProfsTer7***	Insertion (frameshift)	Reported Patients	Sparse brittle hair, sparse eyebrows and eyelashes, conical upper and lower premolar tooth with hypodontia, widely spaced teeth, very dry skin, mildly prominent forehead, periorbital wrinkles
c.358G > T; p.Asp120Tyr	Missense	“Cluzeau 2010[5]”	Dry skin, abnormal nails, palmo-plantar keratoderma
c.335T > G; p.Leu112Arg	Missense	“Bal 2007[4]”	Hypotrichosis, hypodontia, anhidrosis
c.367 G > A; p.Asp123Asn	Missense	“Wohlfart 2016[7]”	Thin, brittle, sparse hair, reduced sweating ability, bilateral amazia, bilateral mature ovarian teratomas, oligodontia
c.391 C > T; p.Pro131Ser	Missense	“Suda 2010[18]”	Only one tooth in the right mandible, sparse hair and eyebrows, normal nails, anhidrosis, extremely low height of the alveolar bone of maxilla and mandible
**c.402-407del; p.Thr135-Val136del**	In-frame deletion	“Chassaing 2010[14]”	Hypotrichosis, hypodontia and anhidrosis, recurrent rhinitis, multiple respiratory infections
**c.570 C > A; p.Asp190Glu**	Missense	“Ahmed 2021[9]”	Sparse hair, hypohidrotic sweating, normal nails, dry skin, no oligodentia

In conclusion, our study identified a novel homozygous insertion variant in *EDARADD*, causing autosomal recessive HED. The segregation analysis strongly supported the recessive inheritance model. Although further studies are needed, our report expands the understanding of the disease, highlighting how genetic testing plays a pivotal role in molecular diagnosis and family risk assessment, also in light of the clinical indistinguishability of the various forms of HED.

### Electronic supplementary material

Below is the link to the electronic supplementary material.

Supplementary Material 1

Supplementary Material 2

## Data Availability

The datasets used and/or analysed during the current study are available from the corresponding author on reasonable request.

## Abbreviations

NFκB: Nuclear factor
ED: Ectodermal dysplasia
HED/AED: Hypohidrotic and anhidrotic ectodermal dysplasias
NMD: Nonsense mediated decay
NGS: Next-generation sequencing
