# Supplementary material for: Novel homozygous frameshift insertion variant in the last exon of the EDARADD causing hypohidrotic ectodermal dysplasia in two siblings: case report and review of the literature
Source: Ital J Pediatr. 2024 Jun 5;50:112. doi: 10.1186/s13052-024-01681-2 (PMC11155060; doi:10.1186/s13052-024-01681-2)
Supplement: Supplementary file 2 — Supplementary Material 2 [file 13052_2024_1681_MOESM2_ESM.docx]

**Novel homozygous frameshift insertion variant in the last exon of the *EDARADD* causing** **Hypohidrotic ectodermal dysplasia in two siblings:** **Case report and review of the literature**

Ahmet KABLAN^1,2^, Elifcan TASDELEN^1,2^

1-Sanliurfa Research and Training Hospital, Department of Medical Genetics, Sanliurfa, Turkey

2-Etlik City Hospital, Department of Medical Genetics, Ankara, Turkey

**Running Title:** **Novel insertion variant in the *EDARADD***

**Ahmet KABLAN:** kablanmd@gmail.com/ORCİD: 0000-0002-8966-2208

**Elifcan TASDELEN:** elifkarakaya2012@gmail.com/ ORCİD: 0000-0003-3917-9792

**Correspondence Address;** Ahmet KABLAN M.D.

Department of Medical Genetics, Etlik City Hospital

Ankara, Turkey, Tel: +905344140906, E-mail: kablanmd@gmail.com

**Abstract**

**Background:** Hypohidrotic ectodermal dysplasia (HED) is a genetic disorder that results in the abnormal development of structures derived from ectodermal tissue. This rare condition predominantly affects the hair, nails, eccrine glands, and teeth. While HED can be caused by various genes, the *EDA, EDAR, EDARADD*, and *WNT10A* genes account for approximately 90% of cases. Notably, HED forms associated with variants in the *EDA, EDAR*, or *EDARADD* genes may exhibit similar phenotypes due to defects in a common signaling pathway. Proper interaction among the products of these genes is crucial for the activation of the nuclear factor (NF-κB) signaling pathway, which subsequently regulates the transcription of targeted genes. The *EDARADD* gene, in particular, harbors one of the rarest reported variants associated with HED.

**Case presentation:** Five-and two-years-old brothers born into consanguineous parents were examined at our outpatient medical genetics clinic at Sanliurfa Training and Research Hospital, Turkey. Both displayed the same classical phenotypic features of HED. The elder had a very sparse dark and brittle hair, sparse eyebrows and eyelashes, conical upper and lower premolar tooth with hypodontia, widely spaced teeth, very dry skin, mildly prominent forehead, and periorbital wrinkles The younger one showed the same, but less severe, clinical features. After thorough examination and patient history evaluation, targeted next generation sequencing analysis yielded the novel homozygous insertion variant c.322_323insCGGGC p.(Arg108ProfsTer7) in *EDARADD.* The mutation has not been reported to date in the literature.

**Conclusions:** In this report, we present two siblings exhibiting classical HED symptoms and a novel insertion variant of the *EDARADD* gene, which leads to a frameshift introducing a stop codon. Both brothers inherited such mutation from their parents, who were heterozygous carriers of the same variant. The present study may shed light about the pathogenic mechanisms underlying HED, and expand the spectrum of *EDARADD* gene variants associated with this condition.

**Keywords:** HED, *EDARADD,* insertion, novel variant, case report

**Background**

Ectodermal dysplasia (ED) is a genetically heterogeneous condition characterized by the abnormal development of structures derived from the ectodermal tissue (1). Among its most frequent forms there are hypohidrotic and anhidrotic ectodermal dysplasias (HED/AED), which present a triad of symptoms, including scalp alopecia or sparse hair (generalized hypotrichosis), teeth anomalies, and hypohidrosis. In addition, some patients may also exhibit minor dysmorphic features such as forehead bumps, rings under the eyes, everted nose, and prominent lips. Several genes have been implicated in the pathogenesis, with the most commonly identified genetic aetiologies being X-linked *EDA* mutations, as well as autosomal *EDAR* and *EDARADD* mutations. Proper function of these three genes and their products is crucial for downstream activation of the nuclear factor (NF-κB), which subsequently regulates the expression of various genes involved in ectodermal development(2).

*EDARADD* is a recently identified gene that encodes a protein with a death domain in the C-terminus, causing HED with an unclear mechanism(3). Indeed, the number of reported variants in *EDARADD* remains relatively small, and the majority of these variants are single nucleotide variations. We report on a novel insertion pathogenic variant causing a frameshift of the *EDARADD* gene(4-7). This variant has been identified, in the homozygous state, in two male siblings born to healthy consanguineous parents. To the best of our knowledge, ours are among the very few patients reported in the literature being affected by HED associated with a pathogenic variant of the *EDARADD* gene.

**Case Presentation**

Five- and two-years-old brothers were born uneventfully, into consanguineous (first degree cousins) parents originating from Syria (**Figure 1b**). They were born uneventfully. Both showed the same classical phenotypic features of HED. The elder brother presented with very dry skin, very sparse dark and brittle hair, sparse eyebrows and eyelashes, mildly prominent forehead, periorbital wrinkles, conical upper and lower premolar tooth with hypodontia, and widely spaced teeth (**Figure 1a**). His developmental milestones and mental status were normal. The younger sibling displayed slightly milder symptoms, with very sparse blonde hair, a mildly prominent forehead, and teeth anomalies similar to those of the elder brother (**Figure 1a**), for which both were receiving dental treatment. The family reported that both brothers have been unable to sweat since birth, and that they had experienced hyperpyrexia due to common infections, successfully managed with medications during hospitalizations. Owing to their reduced ability to sweat, the parents ensured the siblings' protection from sun by sunscreen use. Notably, both parents denied having any relatives with a similar condition, and further examination confirmed that the remaining three living siblings (1 male, 2 females), and the parents themselves, showed no signs of HED.

Informed consent was obtained from the parents. The *EDA, EDAR, WNT10*, and *EDARADD* genes were sequenced through NGS following manufacturer's instructions. Thereafter, Sanger sequencing was conducted, confirming the identified variant, in all family members.

Next-generation sequencing (NGS) analysis did not reveal any causative variants in the *EDA*, *EDAR*, or *WNT10* genes. However, in the *EDARADD* gene (RefSeq accession number NM_145861), a homozygous pentanucleotide insertion, c.322_323insCGGGC (p.Arg108ProfsTer7), leading to a frameshift which introduces a premature stop codon, was identified and considered likely pathogenic in both siblings, based on the American College of Medical Genetics criteria (PVS1-Pathogenic criterion for predicted loss of function variants, PM2-Population data, PP1-Segregation data) . This variant was absent in the Exome Aggregation Consortium and dbSNP database. Subsequent Sanger sequencing was performed in all family members, confirming the homozygosity status in the affected patients. Co-segregation analysis revealed that both parents and one health male sibling were heterozygous carriers of the same mutation (**Figure 1c**).

**Discussion and conclusions**

Ectodermal dysplasia is a genetically and phenotypically heterogeneous condition characterized by the abnormal development of ectodermal derived structures. Although there have been a few other genes reported in individuals with ED-related findings, for hypohidrotic ectodermal dysplasia (HED) four main genes, namely *EDA* (the most common), in addition to *EDAR, WNT10,* and *EDARADD*, have been suggested to be causative(8-11). Neonatologists and paediatricians should raise the suspicion of HED in the presence of congenital skin defects, keeping in mind that such disorder may also be linked with mutations different than those affecting the *EDA* gene, including *EDARADD* (12). Moreover, both recessive and dominant forms of HED caused by *EDARADD* mutations are clinically indistinguishable, and therefore in these cases genetic investigations are decisive for diagnosis confirmation. (3, 7, 13). Indeed, our patients exhibited the triad of symptoms characteristic of HED, including very sparse and brittle hair, teeth anomalies and reduced sweating. Finally, clinicians have to perform a careful differential diagnosis, including conditions sustained by different genetic aetiology such as mutation in *TP63* (14).

In our study, we conducted next-generation sequencing of *EDARADD,* and identified a homozygous variant c.322_323insCGGGC p.(Arg108ProfsTer7) causing a frameshift, and located 5’ upstream of the death domain in exon 6. Such variant has not been previously reported in HED individuals, and data from large population studies is insufficient to assess its frequency. The mutation is an insertion of 5 bases at position c.322, and is expected to impact the protein, made by 215 amino acids in its wild type, introducing a premature termination codon at position 108.

Further studies confirmed co-segregation of the variant in the parents, and identified the heterozygous state in a healthy brother. Based on the segregation analysis, we concluded that this variant causes the recessive form of the disease, as no other family member exhibited symptoms related to HED.

To date, ten different mutations in *EDARADD* have been associated with HED, with only three of these being deletion or insertion (4, 5, 7, 8, 15-19), and different possible molecular pathogenic mechanisms have been hypothesized (disruption of the interaction with EDAR; impairment of the wild-type EDARADD ability to activate NF-kB, disturbance of the multimerization of EDARADD). Additionally, a homozygous gross deletion c.131-?_189+?del was reported with a loss-of-function mechanism.

Zygosity-position-mutation type does not seem to consistently predict the severity of the condition. Published literature is not conclusive in regard to the pathogenic mechanism of *EDARADD*, probably due to very low number of reported variants. However, the loss- of-function mechanism has been considered as a causing process of the disease linked to *EDARADD*(16, 17). This may be due to the loss of protein through nonsense mediated mRNA decay (NMD), leading to the production of truncated proteins that are missing the death domain in exon 6 (highly conserved among species, suggesting its essential function)(20).

Three previous reported variants causing premature termination codons, as shown in **Table 1**, are predicted to go NMD. Since the present variant is not predicted to go NMD but removes more than 10% of transcript, this might be the first truncating variant causing termination codon and not going NMD. Therefore, the predicted molecular pathogenic mechanism is the translation of mRNA containing a premature stop codon, and the production of truncated proteins that are missing the death domain in exon 6. The reported variant is located in the 5’ upstream sequence of the death domain (**Figure 1d**), probably leading to its deficient or absent translation. Also, the phenotypic variability of the syndrome may be explained by different conditions such as modifier genetic variations, possible interactions with regulatory factors and/or the involvement of epigenetic mechanisms in addition to *EDARADD* pleiotropy, which has already been described in the literature regarding other genes(21, 22).

In conclusion, our study identified a novel homozygous insertion variant in *EDARADD*, causing autosomal recessive HED. The segregation analysis strongly supported the recessive inheritance model. Although further studies are needed, our report expands the understanding of the disease, highlighting how genetic testing plays a pivotal role in molecular diagnosis and family risk assessment, also in light of the clinical indistinguishability of the various forms of HED.

**List of abbreviations**

Nuclear factor (NF-κB)

Ectodermal dysplasia (ED)

Hypohidrotic and anhidrotic ectodermal dysplasias (HED/AED)

Nonsense mediated decay (NMD)

Next-generation sequencing (NGS)

**Declarations**

**Ethics approval and consent to participate**

All procedures in this study were in accordance with the ethical standards specified in the World Medical Association Declaration of Helsinki. Ethical approval was not required for this study in accordance with local/national guidelines. Written informed consent was obtained from all participants or if participants are under 16, from a parent and/or legal guardian.

**Consent for publication**

Written informed consent was obtained from the parents of the patients for publication of this case report and accompanying images.

**Availability of data and materials**

The datasets used and/or analysed during the current study are available from the corresponding author on reasonable request.

**Competing Interests**

The authors declare that they have no competing interests

**Funding**

The authors received no financial support for the research, authorship, and publication of this article.

**Authors’ contributions**

AK; acquisition of data, analyzed the clinical data and designed the clinical experiments, designed the experiments, performed PCR, analyzed the sequencing data,

ET; software, validation, wrote the manuscript, conceptualization, interpretation of data, supervised the study, and review the manuscript. All authors read and approved the final manuscript.

**Acknowledgments**

The authors thank to the family for their collaboration in this publication.

**References**

1. Lamartine J. Towards a new classification of ectodermal dysplasias. Clin Exp Dermatol. 2003;28(4):351-5.

2. Mikkola ML. Molecular aspects of hypohidrotic ectodermal dysplasia. Am J Med Genet A. 2009;149A(9):2031-6.

3. Asano N, Yasuno S, Hayashi R, Shimomura Y. Characterization of EDARADD gene mutations responsible for hypohidrotic ectodermal dysplasia. J Dermatol. 2021;48(10):1533-41.

4. Bal E, Baala L, Cluzeau C, El Kerch F, Ouldim K, Hadj‐Rabia S, et al. Autosomal dominant anhidrotic ectodermal dysplasias at the EDARADD locus. Human Mutation. 2007;28(7):703-9.

5. Cluzeau C, Hadj-Rabia S, Jambou M, Mansour S, Guigue P, Masmoudi S, et al. Only four genes (EDA1, EDAR, EDARADD, and WNT10A) account for 90% of hypohidrotic/anhidrotic ectodermal dysplasia cases. Hum Mutat. 2011;32(1):70-2.

6. Headon DJ, Emmal SA, Ferguson BM, Tucker AS, Justice MJ, Sharpe PT, et al. Gene defect in ectodermal dysplasia implicates a death domain adapter in development. Nature. 2001;414(6866):913-6.

7. Wohlfart S, Soder S, Smahi A, Schneider H. A novel missense mutation in the gene EDARADD associated with an unusual phenotype of hypohidrotic ectodermal dysplasia. Am J Med Genet A. 2016;170A(1):249-53.

8. Ahmed HA, El-Kamah GY, Rabie E, Mostafa MI, Abouzaid MR, Hassib NF, et al. Gene Mutations of the Three Ectodysplasin Pathway Key Players (EDA, EDAR, and EDARADD) Account for More than 60% of Egyptian Ectodermal Dysplasia: A Report of Seven Novel Mutations. Genes (Basel). 2021;12(9).

9. Levy J, Capri Y, Rachid M, Dupont C, Vermeesch JR, Devriendt K, et al. LEF1 haploinsufficiency causes ectodermal dysplasia. Clin Genet. 2020;97(4):595-600.

10. Wisniewski SA, Trzeciak WH. A new mutation resulting in the truncation of the TRAF6-interacting domain of XEDAR: a possible novel cause of hypohidrotic ectodermal dysplasia. J Med Genet. 2012;49(8):499-501.

11. Yu M, Fan Z, Wong SW, Sun K, Zhang L, Liu H, et al. Lrp6 Dynamic Expression in Tooth Development and Mutations in Oligodontia. J Dent Res. 2021;100(4):415-22.

12. Piccione M, Serra G, Sanfilippo C, Andreucci E, Sani I, Corsello G. A new mutation in EDA gene in X-linked hypohidrotic ectodermal dysplasia associated with keratoconus. Minerva Pediatr. 2012;64(1):59-64.

13. Monreal AW, Ferguson BM, Headon DJ, Street SL, Overbeek PA, Zonana J. Mutations in the human homologue of mouse dl cause autosomal recessive and dominant hypohidrotic ectodermal dysplasia. Nat Genet. 1999;22(4):366-9.

14. Serra G, Antona V, Giuffre M, Li Pomi F, Lo Scalzo L, Piro E, et al. Novel missense mutation of the TP63 gene in a newborn with Hay-Wells/Ankyloblepharon-Ectodermal defects-Cleft lip/palate (AEC) syndrome: clinical report and follow-up. Ital J Pediatr. 2021;47(1):196.

15. Chassaing N, Cluzeau C, Bal E, Guigue P, Vincent MC, Viot G, et al. Mutations in EDARADD account for a small proportion of hypohidrotic ectodermal dysplasia cases. Br J Dermatol. 2010;162(5):1044-8.

16. Chaudhary AK, Girisha KM, Bashyam MD. A novel EDARADD 5'-splice site mutation resulting in activation of two alternate cryptic 5'-splice sites causes autosomal recessive Hypohidrotic Ectodermal Dysplasia. Am J Med Genet A. 2016;170(6):1639-41.

17. Cluzeau C, Marrakchi S, Picard C, Munnich A, Smahi A, Turki H. First homozygous large deletion in EDARADD gene associated with a severe form of anhidrotic ectodermal dysplasia. J Eur Acad Dermatol Venereol. 2019;33(2):e55-e7.

18. Koguchi-Yoshioka H, Wataya-Kaneda M, Yutani M, Murota H, Nakano H, Sawamura D, Katayama I. Atopic diathesis in hypohidrotic/anhidrotic ectodermal dysplasia. Acta Dermato-Venereologica. 2015;95(4):476-9.

19. Suda N, Bazar A, Bold O, Jigjid B, Garidkhuu A, Ganburged G, Moriyama K. A Mongolian patient with hypohidrotic ectodermal dysplasia with a novel P121S variant in EDARADD. Orthod Craniofac Res. 2010;13(2):114-7.

20. Reyes-Reali J, Mendoza-Ramos MI, Garrido-Guerrero E, Mendez-Catala CF, Mendez-Cruz AR, Pozo-Molina G. Hypohidrotic ectodermal dysplasia: clinical and molecular review. Int J Dermatol. 2018;57(8):965-72.

21. Serra G, Antona V, Schierz M, Vecchio D, Piro E, Corsello G. Esophageal atresia and Beckwith-Wiedemann syndrome in one of the naturally conceived discordant newborn twins: first report. Clin Case Rep. 2018;6(2):399-401.

22. Serra G, Memo L, Antona V, Corsello G, Favero V, Lago P, Giuffre M. Jacobsen syndrome and neonatal bleeding: report on two unrelated patients. Ital J Pediatr. 2021;47(1):147.

**Figure 1. Pictures of affected individuals, pedigree, sequencing display, and representation of reported variants respectively**

**(a)** Dysmorphic features of the proband (above) and affected younger brother (below). Note the very sparse and brittle hair, absent eyebrows and eyelashes, conical teeth with hypodontia, mildly prominent forehead, and periorbital wrinkles
**(b)** Pedigree of the family

**(c)** Sanger sequencing display of the proband and heterozygous mother, flanking sequence of the variant. Black rectangle appoints the position of insertion.
**(d)** Schematic diagram of reported variants. Truncating variants written in red, non-truncating variants in black and reported variant in brown. Protein region encoded by exon 6 magnified and death domain highlighted with different colour. Note the reported variants position in 5’ upstream of death domain in exon 6.
