# Supplementary material for: Novel homozygous frameshift insertion variant in the last exon of the EDARADD causing hypohidrotic ectodermal dysplasia in two siblings: case report and review of the literature
Source: Ital J Pediatr. 2024 Jun 5;50:112. doi: 10.1186/s13052-024-01681-2 (PMC11155060; doi:10.1186/s13052-024-01681-2)
Supplement: Supplementary file 1 — Supplementary Material 1 [file 13052_2024_1681_MOESM1_ESM.docx]

Dear Editor-in-chief,

We would like to thank you and the editor for your and their invaluable efforts to advance the quality of the manuscript for publication. You can find the specific comments and corrections related to the points underlined by the reviewers below.

Editor's comments:

Congratulations to the Authors for their efforts to improve the manuscript, which increases its clarity and fluency.

Final editing indications before publication are the following:

page 5, line 114 "...(p.Arg108ProfsTer7), leading to a frameshift which introduces a premature stop codon, was identified..."

*-revised*

page 7, line 145, change "health" with "healthy"

*-revised*

page 7, lines 161-162, remove from "The loss-of-function" to "EDARADD", and properly locate the references which are present there (i.e. 16 and 17);

*-revised*

page 8, line 163, add "are" before "predicted" and remove from "therefore" to "decay"

-*revised*

line 165, remove the comma after "NMD"

-*revised*

line 169, remove "highly conserved...essential function", as it is a repetition

-*revised*

line 172, add "Also," before the following sentence "The phenotypic variability..."

*-revised*

line 174, remove "and" after "mechanisms".

-*revised*
